# Analgesic effect of thoracic paravertebral block on patients undergoing thoracoscopic lobectomy under general anesthesia

**DOI:** 10.12669/pjms.39.6.7937

**Published:** 2023

**Authors:** Wenhui Zeng, Jianbo Zhang, Leilei Huang, Zhihang Tang

**Affiliations:** 1 Wenhui Zeng, Department of Anesthesiology, The First Affiliated Hospital of Guangzhou University of Chinese Medicine, Guangzhou, Guangdong Province, P.R. China; 2 Jianbo Zhang, Department of Anesthesiology, General Hospital of Southern Theater Command, Guangzhou, Guangdong Province, P.R. China; 3 Leilei Huang, Department of Anesthesiology, The First Affiliated Hospital of Guangzhou University of Chinese Medicine, Guangzhou, Guangdong Province, P.R. China; 4 Zhihang Tang, Department of Anesthesiology, The First Affiliated Hospital of Guangzhou University of Chinese Medicine, Guangzhou, Guangdong Province, P.R. China

**Keywords:** Analgesic effect, General anesthesia, Thoracoscopic lobectomy, Thoracic paravertebral block

## Abstract

**Objective:**

To investigate the analgesic effect of thoracic paravertebral block (TPVB) in patients undergoing thoracoscopic lobectomy under general anesthesia (GA).

**Methods:**

Clinical records of 82 patients who underwent thoracoscopic lobectomy under GA from October 2021 to October 2022 in the General Hospital of Southern Theater Command were retrospectively analyzed. The patients were divided into two groups according to the method of anesthesia used: general anesthesia group (Group-G, n=37), and TPVB plus GA group (Group-T, n=45). The analgesic effect, mean arterial pressure (MAP), heart rate (HR) and the rate of adverse events in both groups were compared.

**Results:**

Visual analogue scale (VAS) scores of patients in Group-T at 12h, 24h and 48h after the operation were significantly lower compared to Group-G (*P*<0.05). MAP and HR the time of tracheal intubation induction (T1), single lung ventilation (T2), skin incision (T3), operation completion (T4), and 20 minutes after the extubation (T5) were lower in both groups compared to T0, and were significantly higher in Group-T compared to Group-G (*P*<0.05). The rate of adverse events in Group-T was 6.67%, significantly lower compared to Group-G (24.32%) (*P*<0.05).

**Conclusions:**

TPVB combined with GA can improve the analgesic effect, improve MAP and HR during the operation, and reduce the incidence of adverse events in patients undergoing thoracoscopic lobectomy.

## INTRODUCTION

Lobectomy is a surgical procedure that removes the entire lobe of the lung and is considered the mainstay of lung cancer treatment. In the past, lobectomy was mainly performed using thoracotomy that is associated with significant trauma and strong stress response.^1^ In recent years, with the continuous development of endoscopic surgery, thoracoscopic lobectomy has gradually become a routine method for surgical treatment of lung cancer patients. It has the advantages of less trauma, fewer complications, and faster recovery compared to thoracotomy. However, this surgical method is still associated with considerable pain.^2^ While most patients undergoing surgery will experience acute postoperative pain, less than half report adequate pain relief.^3^ Acute pain is mainly caused by recent physical injury, and generally lasts no more than two months.^4^ Therefore, increasing anesthesia levels may be sufficient for the adequate acute pain management.

As general anesthesia (GA) is currently the commonly used anesthesia mode for thoracoscopic lobectomy, it is often necessary to increase the dosage of related drugs to meet the requirements of sedation and analgesia during the operation. This may cause significant fluctuations in hemodynamics, and increase the risk of respiratory depression and bradycardia.^5^ In recent years, numerous clinical studies have shown that the injury of the affected side of the chest wall is the main source of pain in thoracoscopic lobectomy. Paravertebral nerve block can obtain unilateral banded pain relief by injecting local anesthetics at the required block plane, blocking noxious stimulation conduction, and achieving good analgesic effect.^6^,^7^ Recently, thoracic paravertebral block (TPVB) combined with GA has become more popular in thoracoscopic lobectomy. However, the data on the application value of this pain control method is still scarce.^8^ While there are studies on the analgesic effect of TPVB, very few focused on TPBV combined with GA. To the best of our knowledge, just one recent study by Feng et al.^9^ on this topic was published up to date.

The purpose of this study was to further analyze the application value of TPVB combined with GA in thoracoscopic lobectomy with the main focus on its analgesic effect and its impact on hemodynamics. Our results may provide reference for the selection of appropriate anesthesia methods for thoracoscopic lobectomy.

## METHODS

Clinical records of 82 patients (47 males and 35 females) who received thoracoscopic lobectomy in the General Hospital of Southern Theater Command from October 2021 to October 2022 were retrospectively selected. Based on the anesthesia approach used, 37 patients received GA and were set as Group-G, and 45 patients received TPVB combined with GA and were set as Group-T.

### Ethical Approval:

This study was approved by the Medical Ethics Committee of General Hospital of Southern Theater Command (No. 2019GCP-0289, Date: 2022-12-06).

### Inclusion criteria:

- Patients who underwent thoracoscopic lobectomy for the first time.
- Patients aged 18-70 years.
- Patients who did not receive chemotherapy or radiotherapy before the operation.
- Patients with American Association of Anesthesiologists (ASA) classification of Grade-I-II.
- Patients with complete medical records.

### Exclusion criteria:

- Patients with serious cardiovascular and cerebrovascular diseases, endocrine diseases, and vital organ dysfunction.
- Patients who underwent other nerve blocks before the operation other than TPVB.
- Patients with thoracic vertebra and thoracic deformity.
- Patients with history of thoracolumbar fracture and surgery.
- Patients with neuropsychiatric or neuromuscular diseases.

Participants fasted for 6-8 hours and did not drink water for two hours before the operation. After entering the operation room, the venous channels were opened, ECG and BIS index were monitored, and radial artery catheterization was routinely performed under local anesthesia to monitor arterial blood pressure. Patients in Group-G received GA as follows: 0.05mg/kg midazolam (Jiangsu Enhua Pharmaceutical Group-Co., Ltd., H10980025) + 0.2mg/kg propofol (Sichuan Kelun Pharmaceutical Co., Ltd. H20203571) + 0.7ug/kg sufentanil (IDT Biologika GmbH, H20100123) + 0.9mg/kg rocuronium (Zhejiang Xianju Pharmaceutical Co., Ltd., H20123188), intravenous injection, followed by placement of double lumen bronchial catheter for mechanical ventilation. The respiratory ratio was controlled at 1:2, the ventilation frequency was set at 12 times/minute, the tidal volume was 6-8ml/kg, and the respiratory membrane carbon dioxide was maintained at 30-40mmHg.

Patients in Group-T received TPBV combined with GA as follows: the patient was positioned in lateral decubitus with his mandible against the chest wall; routine skin disinfection and towel laying was performed, puncture was done under the guidance of color Doppler ultrasound (Sonasite S-Nerve), vertical placement of the spine parallel to the ribs was ensured, the paraspinal space of T4 and T7 was determined and the location of the puncture point was marked. Needle was held in the right hand, and an in-plane puncture was done. Needle penetration into the target space was monitored by ultrasonography. If there was no cerebrospinal fluid, blood and gas backflow, 15ml of 0.5% ropivacaine (Guangdong Jiabo Pharmaceutical Co., Ltd., H20133178) was slowly injected. After 15 minutes, the fine needle was used to evaluate the blocking effect, and general anesthesia induction was started after satisfaction.

### Anesthesia maintenance:

About 1 - 3% sevoflurane (Shanghai Hengrui Pharmaceutical Co., Ltd., H20070172) was administered by inhalation during the operation for anesthesia maintenance. The BIS index was maintained at 40 - 60. Remifentanil (Yichang Humanwell Pharmaceutical Co., Ltd., H20030197) was infused intravenously at 0.05 - 0.2 ug/ (kg · min), and vecuronium bromide (Nanjing Xinbai Pharmaceutical Co., Ltd., H20067267) was injected intermittently to maintain muscle relaxation. During the operation, if the mean arterial pressure (MAP) increased by 20% compared with the basic level, the infusion rate of remifentanil was adjusted appropriately. Ten minutes before the end of the suturing, remifentanil was stopped, 0.1ug/kg sufentanil+5mg tropisetron was injected intravenously (Hainan Lingkang Pharmaceutical Co., Ltd., H20060288), and sevoflurane was administered by inhalation at the end of the operation.

Electronic medical record system, anesthesia record system, rehabilitation room record system and analgesia follow-up system were used to collect all past medical records and relevant information, including demographic indicators (age, body mass index (BMI)). Complications (hypertension, diabetes and coronary heart disease) were recorded. The pain level of patients at 6^th^ hour, 12^th^ hour, 24^th^ hour and 48^th^ hour after operation was recorded. Visual analog scale (VAS) was used to measure the level of pain, with a score of 0-10 points (the higher the score, the more severe the pain).^10^ MAP and HR were recorded and compared at admission (T0), induction of tracheal intubation (T1), one lung ventilation (T2), skin incision (T3), operation completion (T4), and 20 minutes after extubation (T5) in both groups. The adverse events, including bradycardia, hypotension, chills, restlessness, pneumothorax, delayed awakening, nausea and vomiting, were recorded.

### Statistical Analysis:

SPSS26.0 (SPSS Inc., Chicago, IL, USA) was used for analysis. The measurement data of normal distribution were expressed as mean ± standard deviation (*χ̅*±*S*) and compared using Student’s t-test between groups; the non-normal data were expressed as median (IQR) and compared using Mann-Whitney U-test. Multiple time points were compared using repeated measures ANOVA. Number and percentage [n (%)] were used to represent counting data and was compared using *χ^2^*. *P*<0.05 indicated statistical significance.

## RESULTS

A total of 82 patients who underwent thoracoscopic lobectomy were included in this study. Group-G included 37 patients (22 males and 15 females) with the average age of 49.78 ± 10.24 years, and the average BMI of 23.80 ± 2.44 kg/m^2^. ASA classification was as follows: 23 cases were Grade-I, and 14 cases were Grade-II. There were 45 patients in Group-T (25 males and 20 females) with the average age of 51.04 ± 9.67 years, and the average BMI of 22.87 ± 2.05 kg/m^2^. Per ASA classification, 29 cases were classified as Grade-I, and 16 cases were classified as Grade-II. There was no significant difference in the general data between the two groups (*P*>0.05) (Table-I). VAS score was comparable in both groups at 6^th^ hour after the operation. In contrast, VAS score of Group-T was significantly lower than that of Group-G at 12^th^ hour, 24^th^ hour and 48^th^ hour after operation (*P*<0.05) (Table-II). At T0, MAP and HR in both groups were comparable (*P*>0.05). At T1, T2, T3, T4, T5, MAP and HR in the two groups were lower than at T0, and significantly higher in Group-T than in Group-G (*P*<0.05) (Table-III). The incidence of adverse events in Group-T was 6.67%, significantly lower than that in Group-G (24.32%) (*P*<0.05) (Table-IV).

**Table-I:** Compare the basic data of two groups of patients.

Group	n	Gender (n)		Age (year)	Body mass index (kg/m^2^)	ASA grading (n)	
	
		Male	Female			I	II
Gourp-G	37	22	15	49.78±10.24	23.80±2.44	23	14
Gourp-T	45	25	20	51.04±9.67	22.87±2.05	29	16
*χ^2^*/*t*	-	0.126		-0.572	1.873	0.046	
P	-	0.722		0.569	0.065	0.831	

**Table-II:** Compare the VAS scores of the two groups (*χ̅*±*S*, score).

Group	6^th^ hour after operation	12^th^ hour after operation	24^th^ hour after operation	48^th^ hour after operation
Group-G (n=37)	4.00(2.50-5.00)	3.00(2.50-3.00)	2.00(2.00-3.00)	2.00(2.00-2.50)
Group-T (n=45)	3.00(2.00-4.00)	2.00(2.00-3.00)	2.00(2.00-2.00)	2.00(1.00-2.00)
*Z*	-1.919	-2.153	-3.081	-2.142
*P*	0.055	0.031	0.002	0.032

**Table-III:** Comparison of MAP and HR between the two groups (*χ̅*±*S*).

Group	Time	MAP (mmHg)	HR (time/minute)
Group-G (n=37)	T0	104.67±6.08	80.00(77.50-82.50)
	T1	89.89±5.55a	76.00(73.00-78.00)a
	T2	85.45±5.27a	73.00(70.50-75.00)a
	T3	80.37±4.90a	68.00(66.00-70.00)a
	T4	84.37±5.16a	70.00(68.00-72.00)a
	T5	88.32±5.41a	72.00(70.00-74.00)a
Group-T (n=45)	T0	103.11±6.00	81.00(77.50-84.00)
	T1	93.08±5.40ab	79.00(75.50-82.00)a
	T2	87.97±5.20ab	76.00(72.50-79.00)ab
	T3	83.86±5.09ab	72.00(68.50-75.00)ab
	T4	87.02±5.25ab	74.00(70.50-77.00)ab
	T5	90.09±5.24ab	76.00(73.50-80.00)ab

***Note:*** Compared with T0, ^a^ P <0.05; compared with Group-G patients, ^b^P<0.05.

**Table-IV:** Comparison of adverse event rates between the two groups [n (%)]

Group	n	Adverse Events								Total

		Bradycardia	Low blood pressure	Shiver	Restlessness	Pneumothorax	Delayed awakening	Nausea and vomiting	Bradycardia	
Group-G	37	1 (2.70)	1 (2.70)	1 (2.70)	1 (2.70)	1 (2.70)	1 (2.70)	1 (2.70)	2 (5.41)	9 (24.32)
Group-T	45	1 (2.22)	(0.00)	(0.00)	1 (2.22)	(0.00)	(0.00)	(0.00)	1 (2.22)	3 (6.67)
χ^2^	-	-	-	-	-	-	-	-	-	5.068
P	-	-	-	-	-	-	-	-	-	0.024

## DISCUSSION

The results of our study show that TPVB combined with GA is associated with better pain management in patients undergoing thoracoscopic lobectomy compared to GA alone. The combined approach is associated with lower impact on patients’ hemodynamics, and therefore, can potentially reduce the rate of adverse events. The study by Wei W et al^11^ showed that pain associated with the thoracic surgery comes from a wide range of sources, including surgical incision of intercostal nerve conduction and thoracic stimulation. During the operation, pulling and compressing tissues, instrument activities, etc. can stimulate the sympathetic nerve excitability, resulting in significant fluctuations in patients’ blood flow dynamics, higher cardiac load, and myocardial oxygen consumption. Together, these events may increase the incidence of adverse events.

To achieve the required level of analgesia during the surgery under GA, higher doses of anesthetic drugs are needed, which may increase the risk of respiratory inhibition, reduce the quality of recovery, and affect postoperative rehabilitation.^12^ TPVB is based on the injection of local anesthetics in the proximity of the spinal nerve in the intervertebral disc space. Local anesthetics can inhibit the inward flow of Na^+^ and K^+^ in the nerve cell membrane, block the pain signal transmission, block somatic and sympathetic nerves, temporarily block the nerve excitation function of this segment, and inhibit the neurogenic stress response and pain sensation.^13^, ^14^recovery, and to prevent pulmonary complications. So far, no consensus exists on optimal postoperative pain management after VATS anatomic lung resection. Thoracic epidural analgesia (TEA Turhan O *et al*^15^*thoracic paravertebral block* (TPVB compared the effects of three analgesia schemes, such as vertical spinal plane block (ESPB), TPVB, and intercostal nerve block (ICNB), for video-assisted thoracoscopic surgery. In agreement with our results, they reported clear benefits of TPVB with more successful analgesia and less morphine requirement.

MAP and HR can reflect the fluctuation in patient’s hemodynamics. Ozen V *et al*^16^ showed that the use of sedative and analgesic drugs and muscle relaxants during thoracoscopic lobectomy was associated with decreased compensatory ability of patients’ circulatory changes, which can lead to the decrease in MAP and HR. Our results confirm this observation. Large amount of anesthetic drugs, used during surgery with GA, can inhibit neural regulation function, reduce cardiovascular regulation function. Eventually, it may lead to a significant decrease in MAP and HR as a results of the factors, such as decreased blood volume and posture change.^17^ Adding TPVB to GA allows to maintain good analgesic and sedative effects without a potential negative impact on the hemodynamics. TPVB efficiently blocks the lateral cutaneous branch of intercostal nerve, the long thoracic nerve, and the dorsal thoracic nerve, thus reducing surgical stress response and intraoperative hemodynamic fluctuations.^18^

Studies have shown that the increase in the amount of anesthetic drugs used during the operation can lead to adverse events such as bradycardia, hypotension, chills, restlessness, etc.^19^,^20^ In our study, the rate of adverse events in patients received TPVB combined with GA was lower compared to patients who were operated under GA alone. This is consistent with the results of Hu L *et al*, showing that TPVB/ GA combination in patients undergoing thoracoscopic lobectomy effectively reduced the incidence of adverse events, accelerated postoperative recovery, and reduced the length of hospital stay.^21^

### Limitation of the study:

This is a single-center retrospective study with a small sample size, and the participants were not randomly grouped prospectively. Therefore, we cannot rule out the selection bias. However, the characteristics of the participants in the two groups before the surgery were comparable, which supports the validity of our results.

## CONCLUSION

TPVB combined with GA in patients undergoing thoracoscopic lobectomy can effectively improve the analgesic effect, MAP and HR during the operation, reduce the occurrence of adverse events, speed up postoperative recovery, and reduce hospital stay.

### Authors’ contributions:

**WZ** and **JZ:** Conceived and designed the study.

**LH** and **ZT:** Collected the data and performed the analysis.

**WZ** and **JZ:** were involved in the writing of the manuscript and is responsible for the integrity of the study.

All authors have read and approved the final manuscript.
